# Irrigation Management with Brackish Water Impacting Biomass and Protein Productivity in Intercropped *Opuntia stricta* and *Clitória ternatea*

**DOI:** 10.3390/plants15050738

**Published:** 2026-02-28

**Authors:** Rute Maria Rocha Ribeiro, Claudivan Feitosa de Lacerda, Carla Ingryd Nojosa Lessa, Ivo Rabelo de Melo, Jonnathan Richeds da Silva Sales, Kenya Gonçalves Nunes, Maria da Saúde de Sousa Ribeiro, Aiala Vieira Amorim, Marlos Alves Bezerra, Antonia Leila Rocha Neves, Eduardo Santos Cavalcante, Márcio José Alves Peixoto, José do Egito Sales Andrade

**Affiliations:** 1 Departamento de Engenharia Agrícola, Universidade Federal do Ceará, Fortaleza 60455-760, CE, Brazil; rutemaryrocha@gmail.com (R.M.R.R.); ingrydnojosa@alu.ufc.br (C.I.N.L.); instivo01@alu.ufc.br (I.R.d.M.); jonnathanagro@gmail.com (J.R.d.S.S.); sauderibeiro@gmail.com (M.d.S.d.S.R.); aiala.amorim@ufc.br (A.V.A.); educavalcanteufc@gmail.com (E.S.C.); ojosegito@gmail.com (J.d.E.S.A.); 2 Centro de Ciências Agrárias e Biológicas, Universidade Estadual Vale do Acaraú, Acaraú Campus, Acaraú 62040-370, CE, Brazil; kenya_nunes@uvanet.br; 3 Empresa Brasileira de Pesquisa Agropecuária, Fortaleza 60511-110, CE, Brazil; marlos.bezerra@embrapa.br; 4 Secretaria de Desenvolvimento Agrário do Estado do Ceará, Fortaleza 60325-901, CE, Brazil; marcio.peixoto@sda.ce.gov.br

**Keywords:** biosaline agriculture, irrigation, semi-arid region, intercropping, forage production

## Abstract

The use of brackish water associated with intercropping is an approach that can enhance the resilience of agriculture in semi-arid regions. Therefore, this study aimed to evaluate irrigation with brackish water as a strategy to ensure the sustainability of forage production in isolated and intercropped systems. The study was conducted under a hot semi-arid climate in the years 2022, 2023, and 2024. Two water scenarios (rainfed and irrigated) and four production systems with forage cactus-FC (*Opuntia stricta*) and butterfly pea-BP (*Clitória ternatea*) were evaluated: FC—forage cactus, BP—butterfly pea, FC+1BP—forage cactus intercropped with one row of BP, and FC+2BP—forage cactus intercropped with two rows of BP. Butterfly pea received supplemental irrigation from February to August, while the forage cactus was irrigated during the dry season (July to December). Our results showed that the strategic management of irrigation with brackish water optimizes biomass and protein production in crops adapted to the tropical semi-arid region. The FC+1BP intercropping system (forage palm with a row of butterfly pea) proved to be the most advantageous, mainly in terms of crude protein production and water use efficiency, proving to be an alternative for forage production and food security for livestock in the tropical semi-arid region.

## 1. Introduction

Crop productivity in the tropical semi-arid region has been compromised by water scarcity, resulting from the poor temporal and spatial distribution of rainfall, and other abiotic constraints, such as soil and water salinity [1,2]. This results in irregular forage supply for animal feed [3], a problem that may be exacerbated by global climate change [4].

Irrigation is fundamental to producing grains, fruits, and forage in arid and semi-arid regions [5]. However, it is essential to adopt mitigation strategies that allow for sustainable production, such as techniques to increase water use efficiency, crops with lower water requirements, and the adoption of irrigation with alternative water sources, such as brackish and wastewater [6]. Brackish waters contain high-concentration salts, which can affect plant development and alter the physical–chemical properties of the soil [7,8]. However, when used strategically, these water sources can reduce water deficit and increase biomass production without significant salt inputs to the soil [9,10,11].

Among the strategies for using brackish water, supplemental irrigation in rainfed agriculture has shown promising results [6,10]. Supplemental irrigation can be practiced during the rainy season in semi-arid regions, due to the possibility of leaching some of the salts by rainwater [12]. However, to avoid the accumulation of soluble salts in the soil and toxic effects on plants, the use of crops with low water requirements is recommended [11].

Forage cactus (*Opuntia* sp. and *Nopalea cochenillifera*) is an important source of feed for livestock in tropical semi-arid regions, possessing high biomass production, high water use efficiency, high tissue water content, significant mineral concentration, and adequate palatability for animals [13]. Due to its low water requirement (approximately 200 mm annually) and its moderate tolerance to salinity, the forage cactus demonstrates high productive potential with the use of supplemental irrigation with brackish water [12,14]. Furthermore, forage cactus productivity can be significantly increased under deficit irrigation, as demonstrated in other studies [15,16,17]. However, exclusive feeding with forage cactus does not meet the nutritional requirements of animals, making it necessary to combine it with other fiber- and protein-rich foods, such as grasses and legumes [3].

Butterfly pea (*Clitoria ternatea* L.) is a forage legume with low water requirements and tolerance to drought and salinity, able to withstand rainfall regimes of 380 mm per year [18]. This forage crop has a high protein content and can be offered fresh or as hay, with potential for rotational grazing, protein banks, green forage, intercropping, and green manure. It is characterized as a versatile plant with high palatability in all ruminant groups, in addition to excellent nutritional qualities [19].

The intercropping of species with low water requirements, such as forage cactus and butterfly pea, can be an alternative for forage production in semi-arid regions [11,20,21,22]. This production system optimizes land use [23], mainly due to the more effective complementary use of available resources [24]. However, studies involving intercropping systems of cacti with legumes, under brackish water irrigation, are still scarce, since there is no research investigating the influence of intercropping and the use of irrigation with brackish water (full or supplemental) on biomass and protein productivity, comparing monoculture with intercropping.

It is hypothesized that intercropping systems with cacti and legumes irrigated with brackish water will achieve higher values of productivity (biomass and crud protein) and water use efficiency compared to isolated crops. Therefore, this study aimed to evaluate irrigation with brackish water as a strategy to ensure the sustainability of biomass production in isolated and intercropped systems of forage cactus and butterfly pea.

## 2. Results

### 2.1. Soil Salinity

**Figure 1:**
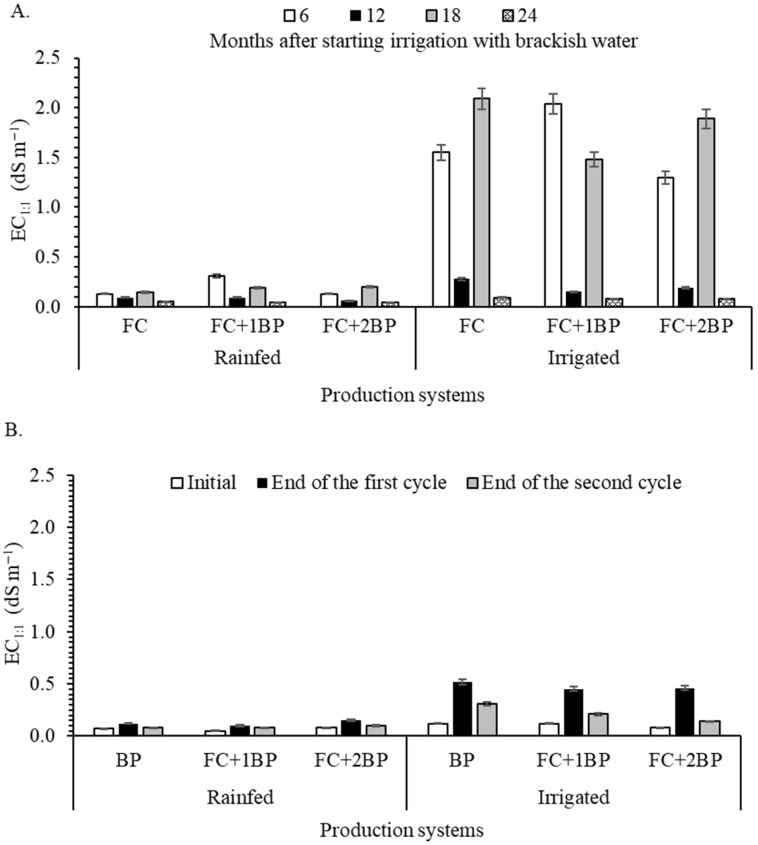
Electrical conductivity of saturated paste in a 1:1 soil ratio for samples collected in areas with forage cactus (**A**) and butterfly pea (**B**), with and without brackish water irrigation. Treatments: FC—forage cactus; BP—butterfly pea; FC+1BP—forage cactus intercropped with a row of butterfly pea; and FC+2BP—forage cactus intercropped with two rows of butterfly pea. Standard error of the mean (n = 4).

Significant differences were observed in the soil EC_1:1_ in forage cactus irrigated with brackish water during the dry season, compared to rainfed treatments. Higher values of EC_1:1_ were observed at 6 and 18 months after the start of treatment differentiation, regardless of the production system (Figure 1A). Otherwise, seasonal rains promoted the leaching of salts, since the soil electrical conductivity values observed in the last evaluation (24 months after the start of irrigation with brackish water) were drastically reduced and reached values close to those found before the experimental trial (Table 1).

A significant increase in soil EC_1:1_ was also observed after the use of supplemental irrigation with brackish water in the root zone of butterfly pea, compared to the rainfed treatment, regardless of the cultivation system (Figure 1B). The higher values of EC_1:1_ were noted at the end of the first cycle in all production systems, especially in the BP (Figure 1B). However, it was observed that the values were much lower than those observed in the forage cactus, due to the lower supplemental irrigation depths applied to butterfly pea.

### 2.2. Biomass Productivity and Protein Concentration

The fresh and dry biomass productivity of forage cactus, obtained at 6, 12, 18, and 24 months after the start of irrigation treatments, was influenced by different water scenarios (*p* < 0.05) and production systems (*p* < 0.01). Irrigation of forage cactus with brackish water during the dry season resulted in an increase of 43.3%, 50.4%, 52.5%, and 21.3% in fresh biomass at 6, 12, 18, and 24 months after the start of irrigation treatments, respectively. Similar results were observed for dry biomass productivity. The FC+2BP production system showed lower fresh and dry biomass productivity at all sampling times (Table 2).

The dry biomass of butterfly pea was significantly influenced by water scenarios and different production systems (Table 3). Irrigation promoted higher biomass productivity only in the first cycle, which is explained by the greater occurrence of dry spells in 2022. Comparing the production systems, it is observed that the productivity of the butterfly pea was lower in the FC+1BP system, largely explained by the lower plant density.

Irrigation with brackish water increased the crude protein concentration in forage cactus and butterfly pea, with increases of 20.2 and 10.7%, respectively, compared to the rainfed treatment (Table 3). Comparing the production systems, the crude protein concentration ranged from 6.0 to 8.7% for forage cactus and from 20.4 to 22.3% for butterfly pea.

### 2.3. Productivity (Biomass and Protein) and Water Use Efficiency for Monocultures and Intercropping Systems

The final productivity of each system was estimated from data obtained with forage palm, 24 months after the start of irrigation with brackish water, and from the two cycles of butterfly pea. The results of the statistical analysis showed the effects of irrigation, production systems, and the interaction between these factors (*p* < 0.01). Irrigation with brackish water at 3–4 dS m^−1^ resulted in increases of 30, 42.5, and 11.5% in dry biomass productivity, crude protein yield, and physical water productivity, respectively, compared to the rainfed treatments (Table 4). The monoculture of butterfly pea showed the lowest values, mainly in terms of dry biomass productivity and water use efficiency. On the other hand, the intercropping of forage cactus with a row of butterfly pea (FC+1BP) stands out with the highest values for these variables, mainly in terms of crude protein productivity.

**Figure 2:**
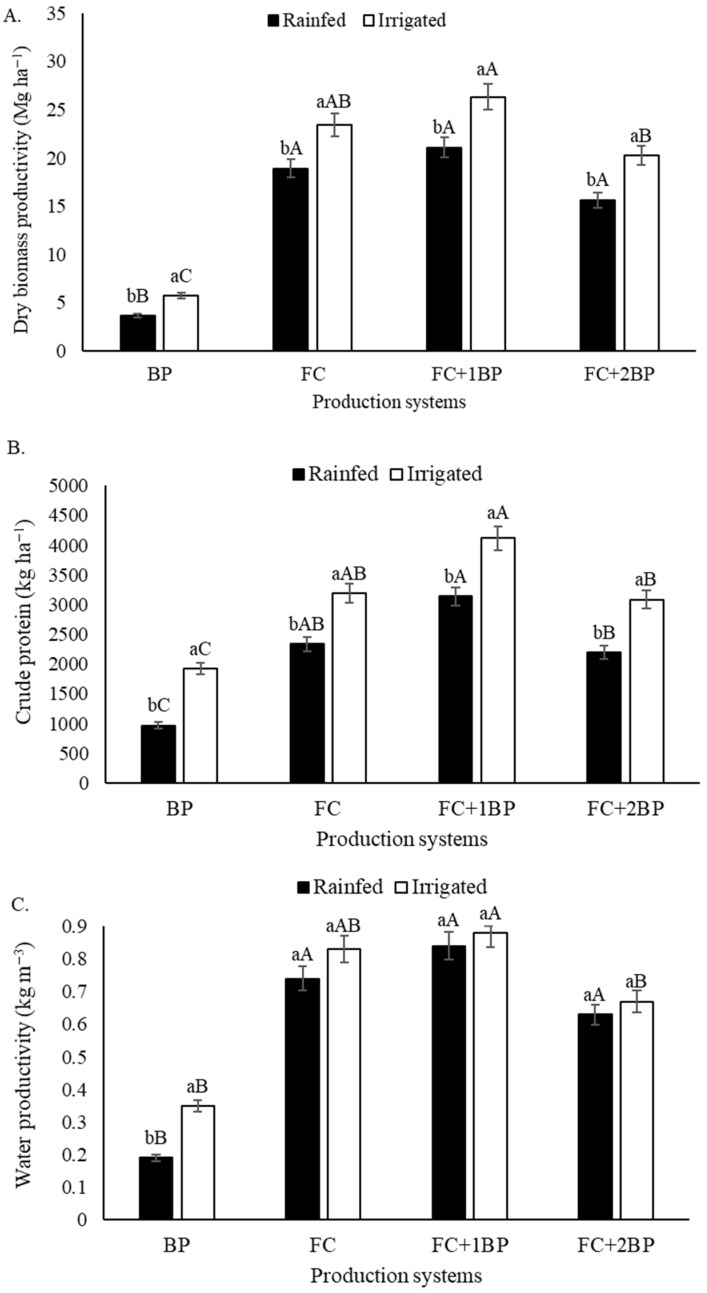
Estimated productivity of dry biomass (**A**), total crude protein (**B**), and physical water productivity (**C**) for the interaction between production systems (monoculture and intercropped systems with forage cactus and butterfly pea) and water treatments (rainfed and irrigated with brackish water). Lowercase letters compare water treatments within each production system; uppercase letters compare production systems within each water treatment. Identical letters do not differ by Tukey’s test (*p* > 0.05).

Analysis of the interaction shows that irrigation with brackish water increased biomass and crude protein productivity, regardless of the system evaluated (Figure 2A,B). On the other hand, irrigation increased the physical productivity of water only in the butterfly pea monoculture, with no difference observed for the forage cactus monoculture and the intercropping systems (Figure 2C). The intercropping of forage cactus with a row of butterfly pea (FC+1BP) proved to be the most advantageous for crude protein production under both rainfed and irrigated with brackish water conditions, achieving the highest values under irrigation.

## 3. Discussion

Irrigation with brackish water in semi-arid regions represents a strategy to mitigate water deficit, with direct implications for the productivity of crops such as forage cactus and butterfly pea, in intercropped and monoculture systems. In our study, this strategy resulted in the accumulation of salt in the soil, especially at the end of the dry season in forage cactus cultivation, but salinization was reversed by the leaching of salt in the rainy season, given the good natural drainage of the soil (Figure 1). These results indicate that there is a low risk of soil salinization when using brackish water irrigation. However, the risks of salinization may be greater when using water with high salinity in soils with high clay content, as highlighted by [6].

The productive response of forage cactus to irrigation with brackish water (3.0–4.0 dS m^−1^) during the dry season shows that water deficit is the main factor limiting biomass production, while salt stress exerts a less pronounced influence. Therefore, the application of irrigation events with waters of moderate salinity favors the productive performance of this crop and contributes to increasing the availability of forage for animal feed, especially during the dry period [25,26].

The positive response of the use of brackish water irrigation on the productive performance of forage cactus (Table 2) demonstrates an alternative to produce forage and the dissemination of biosaline agriculture in tropical semi-arid regions, representing a promising strategy for mitigating the water deficit, as was also demonstrated by [26]. The reduction in the production of fresh and dry biomass of forage cactus under rainfed conditions is related to the water limitation imposed on the plant, which compromises the expansion and emission of new cladodes. This suggests that moderate salinity of the irrigation water is not a limiting factor for this crop, as highlighted by [14].

The water deficit, resulting from the occurrence of dry spells, also proved to be a limiting factor in the production of butterfly pea biomass, especially in the first cultivation cycle (Table 3). This is a result of restrictions on physiological processes, such as stomatal opening and the net photosynthetic rate, which limit the growth of this crop [11]. The increase in dry biomass observed under supplemental irrigation with brackish water indicates that adequate water availability is crucial for tissue growth and the accumulation of dry matter in this legume, even when the water used has moderate salinity, as also observed by [27,28].

Irrigation with brackish water also positively influenced forage quality, reflected in the increase in crude protein concentration, especially in forage cactus (Table 3). Greater water availability can favor increased absorption of nutrients from the soil, especially nitrogen, resulting in greater accumulation of nitrogenous compounds in forage cactus cladodes [29], and favoring the accumulation of crude protein in their tissues [13].

On the other hand, butterfly pea responds to irrigation by investing more energy in the development of the root system, seeking to optimize the absorption of water and nutrients, as observed in ref. [30]. With a denser and deeper root system, there is greater exploitation of water and nutrients in the soil profile, with direct consequences in the increase in nodulation and biological nitrogen fixation [31], resulting in an increase in the crude protein concentration in the leaves.

The presence of butterfly pea in intercropping systems contributed to improvements in soil nitrogen dynamics through the release of root exudates [32]. This may have contributed to the higher protein concentration of forage cactus in intercropped systems compared to monoculture, particularly in the FC+1BP arrangement. This principle has been proven in other intercropped systems, as in the study by [33], who found higher concentrations of crude protein (20% increase) in elephant grass in the system intercropped with butterfly pea compared to those observed in monocultures.

Although butterfly peas have a protein content around 2.8 times higher than forage cactus, the biomass productivity is around 4 times lower. Therefore, the supply of biomass and protein is greatly influenced by crop types and planting density in the intercropped systems. For example, the FC+2BP arrangement resulted in a smaller increase in biomass and protein per hectare, compared to the forage cactus monoculture and the FC+1BP arrangement. The reduction in the plant stand of forage cactus in the FC+2BP system was decisive for the reduction in productive efficiency, as observed in other studies [22,34]. In contrast, the FC+1BP arrangement proved to be more efficient in balancing forage productivity and nutritional quality.

Irrigation with brackish water of 3–4 dS m^−1^ at critical moments for butterfly pea and forage cactus provided the highest estimates of dry biomass productivity, resulting in greater crude protein productivity and high water use efficiency, especially in FC+1BP (Figure 2). The results obtained indicate that salt stress tends to play a secondary role in relation to water deficit, with its influence being modulated by irrigation management and the production system adopted. The absence of negative responses regarding the use of brackish water, combined with the increase in dry biomass production, suggests that water salinity did not have a limiting effect on crop growth, in line with the observations reported by [9]. Convergent results have been reported in other intercropped systems irrigated with brackish water, such as the forage cactus–sorghum, in which complementarity in the use of natural resources by species contributed to increased productivity and resilience of the agricultural systems [18].

The FC+1BP intercropped system showed the best results in terms of crude protein productivity, both under rainfed conditions and irrigated with brackish water (Table 3, Figure 2). The viability of this production system lies in its ability to integrate an energy source (forage cactus) with a protein source (butterfly pea), with the maintenance of high crude protein productivity even in conditions of irrigation with brackish water. This indicates the resilience of this intercropping system. It is inferred that the presence of the butterfly pea legume in the FC+1BP arrangement positively influenced the increase in the available nitrogen content in the soil solution, contributing to the increase in crude protein productivity in this system. According to [35], legumes transfer nutrients to cactus, especially N, through root exudates, root death and nodules. The inclusion of butterfly peas in production systems provides positive characteristics, such as increasing the nutritional value of the forage and fixing N in the system [33].

The high physical water productivity obtained in the FC+1BP production system is quantifiable evidence of the viability of this intercropped system for forage production under a hot semi-arid climate (Figure 2). The greater water productivity observed in the irrigated FC+1BP demonstrates that this arrangement could generate the greatest increase in dry biomass (kg) per cubic meter of water used for irrigation. Integrated production systems promote complementarity in the exploitation of the soil profile, with greater root development and reduced intraspecific competition [13], and generally outperform monocultures in terms of water use efficiency [36].

The butterfly pea (BP) monoculture showed an increase in physical water productivity under supplemental irrigation with brackish water. This behavior indicates that the butterfly pea has high efficiency in converting water into dry biomass when subjected to irrigation with water of marginal quality, reinforcing its productive potential in semi-arid environments. A study developed by ref. [37] also demonstrated the high dry biomass production capacity of butterfly pea when irrigated with brackish water [38]. This highlighted the strong response of butterflies to water availability, reinforcing their efficiency in converting additional water into an increase in dry biomass, when the water deficit is mitigated. Although the butterfly pea monoculture showed an increase in the physical water productivity under brackish irrigation, the FC+1BP intercropping proved to be more balanced by integrating biomass productivity, nutritional quality, and water use efficiency.

## 4. Materials and Methods

### 4.1. Location and Characterization of the Area

**Figure 3:**
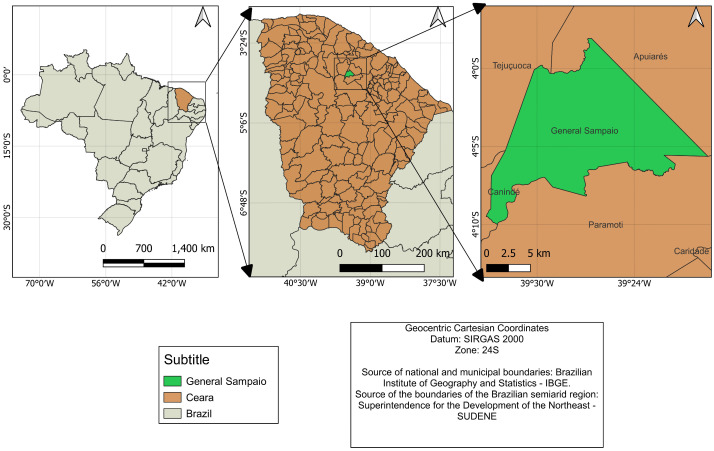
Location of the experimental area in the municipality of General Sampaio, Ceará, Brazil.

The experiment was conducted from February 2022 to August 2024 in the municipality of General Sampaio (4°03′10″ S; 39°27′16″ W, 93 m), Ceará, Brazil (Figure 3). The local climate is hot semi-arid, with predominant rainfall from January to April and an average temperature of 26 to 28 °C [39].

**Figure 4:**
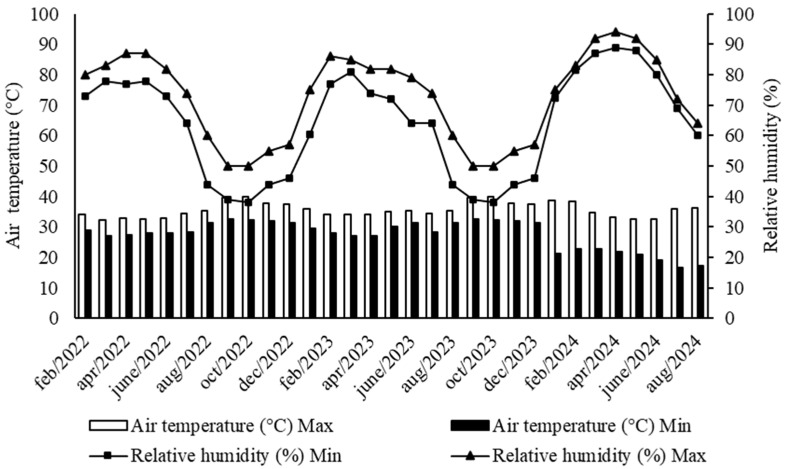
Maximum and minimum air temperature and relative humidity during the experimental period. Source: Data from Automatic Station No. 186 of Tejuçuoca—Ceará, Brazil, extracted from the website of the Ceará Foundation for Meteorology and Water Resources (FUNCEME).

The temperature and relative humidity data during the experiment are shown in Figure 4. The data were obtained from an automatic weather station located approximately 20 km from the experimental area.

Soil samples were previously collected from the 0 to 0.20 m layer for chemical and granulometric analyses, which were performed according to the methodology of [40]. The soil used in this study was characterized as Planosol according to the Brazilian Soil Classification System [41], equivalent to the order Alfisols in the American Soil Taxonomy [42]. Its chemical and granulometric characteristics are presented in Table 1.

Soil liming was carried out in January 2022, with an application of 2.82 t ha^−1^, according to Fernandes [43]. Dolomitic limestone was applied to the surface of the moist soil, then incorporated by harrowing to a depth of 0.20 m.

### 4.2. Experimental Design and Treatments

The experimental design was a randomized complete block design with split plot arrangement and four replications. The main plots corresponded to two water regimes: rainfed and irrigated; the subplots consisted of four biomass production systems: FC—forage cactus (plant spacing of 2.0 m × 0.1 m between rows and plants, respectively, plant density of 50,000 plants ha^−1^); BP—butterfly pea (1.0 × 0.1 m, 100,000 plants ha^−1^); FC+1BP—forage cactus (2.0 × 0.1 m, 50,000 plants ha^−1^) intercropped with a row of butterfly pea (1.0 × 0.1 m, 50,000 plants ha^−1^); and FC+2BP—forage cactus (3.0 × 0.1 m, 33,333 plants ha^−1^) intercropped with two rows of butterfly pea (1.0 × 0.1 m, 66,666 plants ha^−1^). Each experimental unit had an area of 54 m^2^, with a total area of 0.2 hectares.

### 4.3. Crops and Growing Conditions

The crops used were forage cactus (*Opuntia stricta* (Haw) Haw), cultivar Mexican Elephant Ear, and the legume butterfly pea (*Clitória ternatea* L.). For planting the cactus grove, seed cladodes were provided by the Secretariat of Agrarian Development of the State of Ceará, originating from the Lagoa de São Miguel farm, municipality of Quixeramobim—CE (5°11′56″ S, 39°17′34″ W, 250 m), and from a producer with a national registration of seeds and seedlings with the Ministry of Agriculture and Livestock of Brazil. Seeds of butterfly pea were obtained from the germplasm bank of the Seed Laboratory of the Federal University of Ceará.

The planting of forage cactus was carried out in February 2022, with two-thirds of cladodes immersed in the soil to ensure firmness and better development. The planting of the butterfly pea was carried out in February 2022 and 2023. Seeds of butterfly pea were subjected to mechanical scarification with sandpaper and immersion in warm water at 80 °C for 12 h to break dormancy, using approximately 30 seeds per linear meter. One week after sowing, thinning was carried out, leaving 10 plants per linear meter.

Fertilization practices were carried out uniformly for all production systems, according to technical recommendations for forage cactus (30 tons of bovine manure ha^−1^ year^−1^) [44] and for butterfly pea (50 kg ha^−1^ of P_2_O_5_ plus 60 kg ha^−1^ of K_2_O) [38,45].

Localized drip irrigation was used, with one line per row of plants, using flexible polyethylene drip tapes with a flow rate of 1.7 L h^−1^, emitter spacing of 0.20 m, operating pressure of 101.32 kPa, and a distribution uniformity coefficient of 90%. For butterfly pea, supplemental irrigation was used during the dry spells (five days without rain) between February and August (encompassing the region’s rainy season) in both crop cycles (2022 and 2023). The forage cactus was irrigated only during the dry season, between July and December (2022 and 2023), every seven days. The two crops were irrigated simultaneously only in the months of July and August, but on different days of the week. The drip irrigation system was also controlled separately for each crop.

The irrigation depth was adjusted to the volume of water available on site and the water requirements based on crop coefficient for each species [27,46], determining the volume of water for each plant. The irrigation rate was calculated from the volume applied per plant in relation to the area covered. The total supplemental irrigation depth for butterfly pea during the first cycle (2022) was 121.8 mm, with 37.5 mm applied up to the 1st cut and 84.7 mm between the first and second cuts. In the second cycle (2023), the total water depth applied was 112.8 mm, with 28.1 mm applied up to the 1st cut and 84.7 mm up to the 2nd cut. For the forage cactus, irrigation depths of 254.4 mm and 281.2 mm were applied during the dry season of 2022 and 2023, respectively.

The irrigation water was obtained by mixing water from a deep well (70 m deep) and a shallow well (10 m deep). The average electrical conductivity of the irrigation water (mixture of the two water sources) was 3.0 and 4.0 dS m^−1^ for butterfly pea and forage cactus, respectively, according to the sensitivity of these two crops to water salinity, as per [11,14]. The water mixture ratio was adjusted to maintain these values over time.

**Figure 5:**
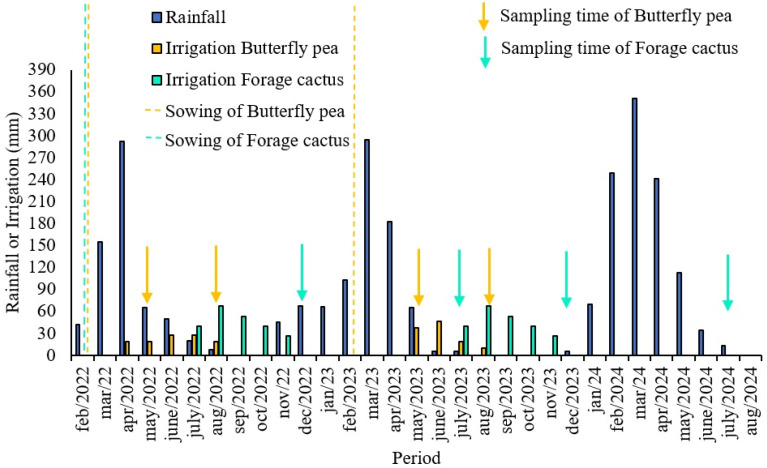
Irrigation depths provided by precipitation and irrigation during the experimental period. Dashed lines indicate planting dates and arrows indicate sampling dates.

Figure 5 shows the water depth supplied by rainfall and irrigation during the experimental period. Rainfall data were collected daily using a rain gauge installed near the experimental area.

### 4.4. Soil Analysis

Soil samples were collected from the planting rows in the 0–0.20 m layer to evaluate the electrical conductivity of the soil:water 1:1 extract (EC_1:1_) [47]. The samplings were carried out before and after the dry and rainy seasons, in the years 2022, 2023 and 2024.

### 4.5. Crop Yield and Water Use Efficiency

The productivity of forage palm was evaluated on samples composed of five plants, collected at 6 (December 2022), 12 (July 2023), 18 (December 2023), and 24 months (August 2024) after the start of irrigation with brackish water, using July 2022 as a reference month. The cutting was done leaving only the basal cladode of each plant, with the cladodes separated into primary, secondary, and tertiary. Fresh and dry biomass data were expressed in mg ha^−1^.

To obtain fresh and dry biomass production of butterfly pea, a sample of thirty plants per treatment was cut at a cutting height of 0.10 m. The first cut was performed at 120 DAP (days after planting) and the second cut 60 days after the first, in the years 2022 and 2023 (Figure 5), totaling two cycles of this crop. The values were expressed in mg ha^−1^.

The dried samples of the two crops were ground in a Willey-type mill. The extract was obtained by wet digestion with sulfuric acid (H_2_SO_4_). The nitrogen content was determined by the semimicro Kjeldahl method [48]. The percentage of crude protein was calculated according to the methodology of [49].

Crude protein productivity was obtained by multiplying the percentage of crude protein by the total dry biomass productivity of each crop. The protein productivity of the intercropping systems (FC+1BP, and FC+2BP) was obtained by summing the protein productivity of the two crops. The production values from the last palm harvest in August 2024 and the sum of the two cycles of the butterfly pea were considered. The values were expressed in kg ha^−1^.

Water use efficiency was assessed using the physical water productivity indicator (kg m^−3^), estimated by the ratio between dry biomass productivity and the volume of water (irrigation + precipitation) applied from February 2022 to August 2024, according to [5]. For the estimation of this indicator, the dry biomass productivity obtained by summing the production of the two butterfly pea cycles and the production value referring to the last cut of the forage cactus were considered.

### 4.6. Data Analysis

The data were subjected to the Shapiro–Wilk normality test (*p* < 0.05). Subsequently, analysis of variance was performed, and the means were compared using Tukey’s test (*p* < 0.05). Statistical analyses were performed using R studio software version 4.4.1 [50].

## 5. Conclusions

Our study highlights the potential of brackish groundwater as a strategy for mitigating water deficit, optimizing water use and increasing crude protein productivity in forage crops, such as forage cactus and butterfly pea, with low risks of soil salinization. The FC+1BP intercropping system (forage palm with a row of butterfly pea) proved to be the most advantageous, mainly in terms of crude protein production and water use efficiency, proving to be an alternative for forage production and food security for livestock in the tropical semi-arid region.

Despite the low risk of soil degradation, further studies are recommended to assess the risks of salinization in soils with different physical characteristics, since maintaining soil quality is fundamental to the sustainability of biosaline agriculture. Furthermore, there is a need for new studies that consider greater diversification of forage cactus intercropping, including other legumes with higher biomass productivity than butterfly pea.

## Figures and Tables

**Table 1:** Chemical and granulometric characteristics of the soil of the experimental area.

Depth (cm)	K^+^	Ca^2+^	Mg^2+^	SB	Na^+^	H+Al	CEC	P	pH	EC_1:1_	ESP
	cmol_c_ dm^−3^							mg kg^−1^	H_2_O	(dS m^−1^)	%
0–20	0.22	0.51	0.27	1.0	0.19	0.8	1.8	3.1	5.6	0.29	10.0
Depth (cm)	Sand		Silt			Clay			Soil texture		
	(%)										
0–20	85.4		8.5			6.1			Sandy		

Potential of hydrogen (pH), electrical conductivity of the soil:water 1:1 extract (EC_1:1_), Sum of bases (SB), Cation exchange capacity (CEC), Exchangeable sodium percentage (ESP).

**Table 2:** Fresh biomass productivity and dry biomass productivity of forage cactus in monoculture and intercropped systems with butterfly pea, under rainfed and irrigated with brackish water.

	Fresh Biomass (mg ha^−1^)				Dry Biomass (mg ha^−1^)			
	Months After Starting Irrigation with Brackish Water							
Treatments	6	12	18	24	6	12	18	24
Rainfed	18.88 b	63.26 b	96.30 b	145.35 b	1.93 b	6.32 b	10.96 b	16.50 b
Irrigated	27.01 a	95.15 a	146.87 a	176.32 a	2.63 a	9.51 a	16.63 a	19.90 a
FC	27.17 a	91.90 a	132.00 a	180.55 a	2.69 a	9.19 a	15.45 a	21.16 a
FC+1BP	26.10 a	91.48 a	144.00 a	176.11 a	2.56 a	9.14 a	16.10 a	19.62 a
FC+2BP	15.56 b	54.23 b	88.77 b	125.85 b	1.59 b	5.42 b	9.83 b	13.82 b
CV WS (%)	19.07	14.12	5.49	7.95	18.44	14.12	7.26	6.58
CV PS (%)	19.84	16.35	21.25	14.57	24.75	16.35	22.32	13.6

Coefficient of variation (CV); Water scenarios (WS), Production systems (PS). Identical letters in the columns, for water regimes or cropping systems, do not differ by Tukey’s test (*p* > 0.05).

**Table 3:** Productivity of dry biomass of butterfly pea and percentage of crude protein of forage cactus and butterfly pea in monoculture and intercropped systems, under rainfed and irrigated with brackish water.

	Dry Biomass Butterfly Pea (mg ha^−1^)		Crude Protein (%)	
	Cycle		Crops	
Treatments	First	Second	Forage Cactus	Butterfly Pea
Rainfed	1.45 b	1.66 a	6.97 b	19.81 b
Irrigated	3.23 a	1.64 a	8.88 a	22.18 a
FC	-	-	6.01 b	-
FC+1BP	1.51 b	1.30 b	7.56 ab	20.43 b
FC+2BP	1.66 b	1.91 a	8.69 a	20.51 ab
BP	3.85 a	1.74 a	-	22.26 a
CV WS (%)	20.8	14.45	8.56	8.82
CV PS (%)	25.4	16.1	6.76	6.08

Coefficient of variation (CV); Water scenarios (WS), Production systems (PS). Identical letters in the columns, for water regimes or cropping systems, do not differ by Tukey’s test (*p* > 0.05).

**Table 4:** Estimated productivity of dry biomass, total crude protein, and physical water productivity for monoculture and intercropped systems with forage cactus and butterfly pea, under rainfed and irrigated with brackish water.

Treatments	Dry Biomass (Mg ha^−1^)	Crude Protein (kg ha^−1^)	Water Productivity (kg m^−3^)
Rainfed	19.6 b	2160.3 b	0.61 b
Irrigated	24.8 a	3079.7 a	0.68 a
BP	5.6 c	1448.4 c	0.27 c
FC	21.2 ab	2763.4 b	0.77 a
FC+1BP	22.4 a	3626.5 a	0.86 a
FC+2BP	17.1 b	2641.5 b	0.65 b
CV WS (%)	9.90	8.31	9.84
CV SP (%)	15.38	17.94	14.82

Coefficient of variation (CV); Water scenarios (WS), Production systems (PS). Identical letters in the columns, for water regimes or cropping systems, do not differ by Tukey’s test (*p* > 0.05).

## Data Availability

The original contributions presented in this study are included in the article.
